# The effect of ligand amount, affinity and internalization on PSMA-targeted imaging and therapy: A simulation study using a PBPK model

**DOI:** 10.1038/s41598-019-56603-8

**Published:** 2019-12-27

**Authors:** Nusrat J. Begum, Gerhard Glatting, Hans-Jürgen Wester, Matthias Eiber, Ambros J. Beer, Peter Kletting

**Affiliations:** 1 Medical Radiation Physics, Department of Nuclear Medicine, Ulm University, Ulm, Germany; 2 Department of Nuclear Medicine, Ulm University, Ulm, Germany; 3 Technical University of Munich, Pharmaceutical Radiochemistry, Munich, Germany; 4 Technical University of Munich, School of Medicine, Klinikum rechts der Isar, Department of Nuclear Medicine, Munich, Germany

**Keywords:** Targeted therapies, Computational models, Pharmacokinetics, Computational science, Predictive medicine

## Abstract

The aim of this work was to investigate the effect of ligand amount, affinity and internalization of prostate-specific membrane antigen (PSMA)-specific ligands on the activity concentrations for PET/CT imaging and on the absorbed doses for therapy. A physiologically-based pharmacokinetic (PBPK) model for PSMA-specific ligands was implemented. Thirteen virtual patients with metastatic castration-resistant prostate cancer were analysed. Simulations were performed for different combinations of association rates *k*_*on*_ (0.1–0.01 L/nmol/min), dissociation rates *k*_*off*_ (0.1–0.0001 min^−1^), internalization rates *λ*_*int*_ (0.01–0.0001 min^−1^) and ligand amounts (1–1000 nmol). For imaging the activity was normalized to volume and injected activity (^68^Ga-PSMA at 1 h). For therapy the absorbed dose was calculated for 7.3 ± 0.3 GBq ^177^Lu-PSMA. The effect of the investigated parameters on therapy were larger compared to imaging. For imaging, the combination of properties leading to the highest tumour uptake was *k*_*on*_ = 0.1 L/nmol/min, *k*_*off*_ = 0.01 min^−1^ for typical ligand amounts (1–10 nmol). For therapy, the higher the internalization rate, the larger was the required ligand amount for optimal tumour-to-kidney ratios. The higher the affinity, the more important was the choice of the optimal ligand amount. PBPK modelling provides insight into the pharmacokinetics of PSMA-specific ligands. Further *in silico* and *in vivo* studies are required to verify the influence of the analysed parameters.

## Introduction

Theranostics refers to the use of individual patient-level biological information from imaging to determine an optimal therapy for an individual patient^1,2^. Thus, ideally, theranostic agents can be used for both, imaging and therapy to minimize changes in pharmacokinetics due to different chemical structures. The prostate-specific membrane antigen (PSMA) has received increasing interest for theranostic approaches in prostate cancer^3,4^. PSMA-specific ligands are predominantly labelled with ^68^Ga/^177^Lu or ^18^F/^177^Lu for imaging and therapy^5^.

Beside important ligand properties like molecular size or lipophilicity, the association and dissociation rate *k*_*on*_ and *k*_*off*_ and the internalization rate *λ*_*int*_ influence the pharmacokinetics^6^. The binding affinity is described by the dissociation constant *K*_*D*_ = *k*_*off*_/*k*_*on*_^7^. High affinity (i.e. low *K*_*D*_) is a prerequisite for imaging and therapy especially for small molecules with fast clearance^8^. However, it is unclear whether ligands optimized for imaging are also ideal candidates for radionuclide therapy, because of different quantities of interest (activity concentrations vs absorbed doses) and different administered ligand amounts^9^. Basic work on the influence of affinity and ligand amount has been conducted^10,11^. However up to now no investigation considering PSMA-targeting small molecules, internalization, different combinations of the association and dissociation rate *k*_*on*_ and *k*_*off*_ and various tissues for both imaging and therapy concurrently has been performed.

*In silico* investigations, e.g. by means of simulation and modelling, assist in the development and optimization of theranostics^12^. Computational approaches, e.g. using physiologically based pharmacokinetic (PBPK) models are important tools for the development of accurate and personalized treatments that are both cost- and time-effective^12^. PBPK models are increasingly used to systematically investigate pharmacokinetic parameters and absorbed doses^13–16^. PBPK models consider the individual patient biokinetics in the organs at risk (OARs) and in tumours^17^. Recently a whole-body PBPK model has been developed for PSMA radioligand therapy^15^ based on data from PET/CT imaging with ^68^Ga-PSMA-11 and peri-therapeutic measurements with ^177^Lu-PSMA I&T.

The aim of this work was, to investigate the interconnected effect of affinity, internalization and injected ligand amount of PSMA-specific ligands using a PBPK modelling and simulation approach. For imaging the normalized activity concentrations in tumour, background, and OARs and for therapy the absorbed doses of tumours and the OARs were determined. These quantities were investigated for different combinations of ligand amounts (1–1000 nmol), internalization rates *λ*_*int*_ (0.01, 0.001 and 0.0001 min^−1^) and dissociation constant *K*_*D*_ values (1, 0.1 and 0.01 nM).

## Materials and Methods

### Patient individualized PBPK model

A recently published PBPK model based on data from PET/CT imaging with PSMA-11 and peri-therapeutic measurements with PSMA I&T^15^ was implemented in Simbiology/MATLAB (MATLAB R2018a, The MathWorks, Inc). The model includes all physiologically and physically relevant mechanisms such as blood flow, plasma protein binding, PSMA-specific binding, internalization and release from the cells, excretion, and physical decay. An effective internalization rate as reported for antibody pharmacokinetic modelling^18,19^ was assumed. The number of PSMA receptors (which were estimated in previous work using PSMA-11 and PSMA I&T data) also represent effective values including all receptor subtypes.

The competition of labelled and unlabelled peptide (with the same affinity) is described by two separate circulation systems for binding to PSMA and by physical decay. The tumours, kidneys, liver, and the gastrointestinal tract were considered as PSMA-positive tissues. Two tumour lesions were explicitly modelled showing highest uptake and no overlap with other PSMA-positive tissues. All other tumour lesions were merged into tumour REST. The cumulated activity and volume of the tumour REST were obtained by adding all lesions slice by slice using isocontours of 15–20%. To correct the overestimation or underestimation of tumour volume, a correction factor was estimated in the fitting process^15^.

For the kidney model, PSMA-specific binding, internalization, release and all mechanisms pertaining to clearance were included. Amino acids were administered to block unspecific uptake as applied in PRRT. Therefore, unspecific uptake in kidney was assumed to be low. The model, its parameters and the fitting methods are described in detail elsewhere^15,16^. In brief, the model parameters were fitted to time-activity data of thirteen patients including covariates such as age and body weight^15^. These 13 patient-individualized models (virtual patients) were used in this work as a basis for all simulations. The parameter distributions of the tumour were wide, e.g. total tumour volume (Median: 0.4, Range: 0.02–5) l, receptor densities (Median: 50, Range: 4–124) nmol/l, blood flows (Median: 0.2, Range: 0.02–1.6) ml/min/g. Receptor densities in tumour REST were higher and perfusion lower on average compared to the single lesions. The virtual patients account for all these differences. The institutional review board of the Technische Universität München approved all procedures and the compassionate use of ^177^Lu-PSMA I&T in metastatic castration-resistant prostate cancer patients who had no other therapeutic options. All procedures in this study were performed in accordance with relevant guidelines and regulations. All subjects signed a written informed consent form.

### Simulations

The simulations were conducted for dissociation constant *K*_*D*_ values of 1, 0.1 and 0.01 nM and ligand amounts of 1–1000 nmol inr of $\sqrt{10}$ with an injected mean activity (±SD) of (7.3 ± 0.3) GBq ^177^Lu-PSMA. The range of the herein used association and dissociation rate *k*_*on*_ and *k*_*off*_ values were taken from in-house surface plasmon resonance measurements^20,21^. For example, for the ligand PSMA-617 dissociation constant *K*_*D*_ (*k*_*off*_/*k*_*on*_) was determined to be 0.06 nM. The dissociation rate *k*_*off*_ value of 0.0001 min^−1^ (not measured) was added for a more systematic investigation. Different combinations of association and dissociation rates *k*_*on*_ and *k*_*off*_ for the same dissociation constant *K*_*D*_ value were investigated. To limit the number of possible combinations of association and dissociation rates *k*_*on*_ and *k*_*off*_ to a reasonable number, two combinations for each dissociation constant *K*_*D*_ were investigated for 3 different internalization rate *λ*_*int*_ values (0.01, 0.001 and 0.0001) min^−1^ (Table 1).

**Table 1:** Investigated combinations of *k*_*on*_ and *k*_*off*_.

*K*_*D*_^a^ [nM]	*k*_*off*_^b^ [min^−1^]	*k*_*on*_^c^ [L/nmol/min]
0.01	0.0001	0.01
0.01	0.001	0.1
0.1	0.001	0.01
0.1	0.01	0.1
1	0.01	0.01
1	0.1	0.1

^*a*^*K*_*D*_ = Dissociation constant (*K*_*D*_ = *k*_*off*_/*k*_*on*_); ^*b*^*k*_*off*_ = Dissociation rate; ^*c*^*k*_*on*_ = Association rate.

### Imaging

The normalized activity concentrations without decay correction 1 h after injections of ^68^Ga-PSMA were investigated for two tumour lesions, tumour REST, background (including muscle and fat), organs at risk OARs (kidneys, liver, red marrow), gastrointestinal tract and lung. The normalized activity concentrations *c*_*i*_ (*t*) of each relevant organ *i* were calculated using (Eq. 1), where *A*_*i*_(*t*) is the activity in organ *i*, *A*_0_ is the injected activity and *V*_*i*_ is the volume of organ *i*:

1 $${c}_{i}(t)=\frac{{A}_{i}(t)}{{A}_{0}\cdot {V}_{i}}$$

### Therapy

The absorbed doses of ^177^Lu-PSMA radioligand therapy (Activity: (7.3 ± 0.3) GBq) were calculated for therapeutically relevant organs *i*, i.e. the two tumour lesions, the tumour REST, the kidneys and red marrow, based on the MIRD formalism as follows:

2 $${D}_{i}(T)={A}_{0}\cdot {\tilde{a}}_{i}(T)\cdot {S}_{i\leftarrow i}$$

with the dose *D*_*i*_ (*T*) to organ *i* (where *T* = 30000 min), the injected activity *A*_0_, the time-integrated activity coefficient ${\tilde{a}}_{i}(T)$ and the dose factor organ *i* to organ *i* ${S}_{i\leftarrow i}$. For tumours and kidneys, only the self-dose was considered. The S-values for each tumour lesion and OARs were determined based on the data of OLINDA/EXM for ^177^Lu for spheres^15^. For bone marrow the absorbed dose calculation includes other relevant organs and tumours^15^.

## Results

### Imaging

**Figure 1:**
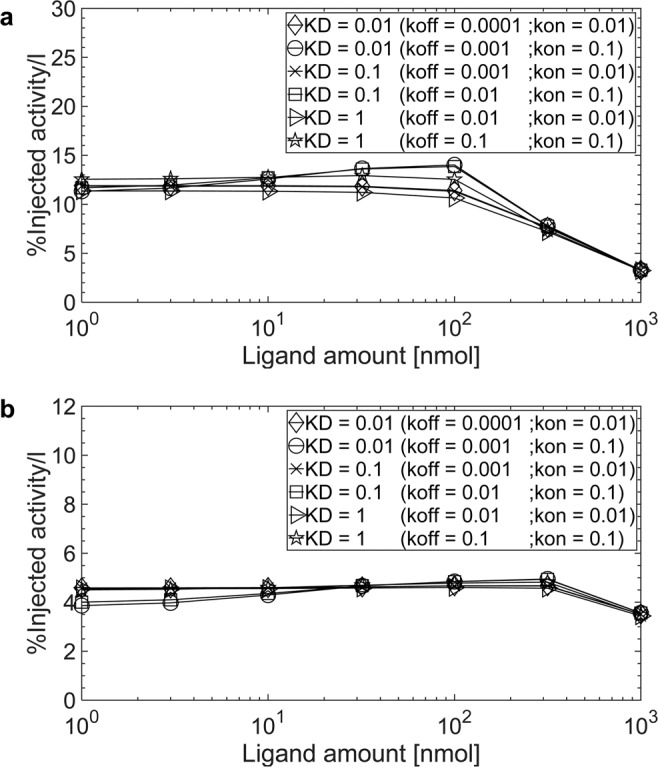
Normalized activity concentrations for ^68^Ga-labelled PSMA-specific ligands (1 h p.i.) for the internalization rate λ_int_ = 0.001 min^−1^ in (**a**) a tumour lesion (receptor density 33 nmol/l and perfusion 0.08 ml/g/min) and (**b**) tumour REST (receptor density 66 nmol/l and perfusion 0.04 ml/g/min) of patient 5.

The results for imaging considering all patients, different ligand amounts, association and dissociation rates *k*_*on*_ and *k*_*off*_, and internalization rates *λ*_*int*_ are presented in the supplement (Table S1 for tumour and Table S2 and S3 for organs at risk OARs and background). Figure 1a,b show the normalized activity concentration in a tumour lesion and tumour REST depending on association and dissociation rates *k*_*on*_ and *k*_*off*_, and the applied ligand amount for a typical patient. The figure for the background is provided in the supplement (Fig. S1). All results described in the sections below refer to the internalization rate *λ*_*int*_ = 0.001 min^−1^ which is used in the published model^15^.

#### Effect of association and dissociation rate k_on_ and k_off_

Tumours. In general the effect of the investigated combinations of association and dissociation rates *k*_*on*_ and *k*_*off*_ on the normalized activity concentration 1 h p.i. is low (Fig. 1). However, varying the association rate *k*_*on*_ seems to have a larger effect than varying the dissociation rate *k*_*off*_. Changing the association rate *k*_*on*_ from 0.01 to 0.1 L/nmol/min (with a fixed dissociation rate *k*_*off*_ of 0.01 min^−1^) increased the normalized concentration by a factor of 1.2 ± 0.3 for tumour lesions and 1.1 ± 0.1 for tumour REST using the commonly used ligand amount of 10 nmol for imaging (Supplement, Table S4).

Organs at risk and background. Similar to the tumour, the association rate *k*_*on*_ has a more pronounced effect on the normalized activity concentrations in OARs than the dissociation rate *k*_*off*_. Decreasing the dissociation constant *K*_*D*_ by increasing the association rate *k*_*on*_ led to an increase of the normalized activity concentration in the kidneys and liver, and to a decrease in the lung, gastrointestinal tract, red marrow and background (Supplement, Tables S2 and S3).

#### Effect of ligand amount

Tumours. Overall the effect of ligand amount (1–32 nmol) is low. However, the effect of the ligand amount is more pronounced for the dissociation constant *K*_*D*_ < 1 nM with an association rate *k*_*on*_ = 0.1 L/nmol/min. For combinations of association and dissociation rates *k*_*on*_ and *k*_*off*_ leading to the same dissociation constant *K*_*D*_, the higher the association rate *k*_*on*_ the more important is the injected ligand amount (Fig. 1a,b). The maximal improvements were achieved if the ligand amount of 32 nmol is administered. The highest improvement was obtained with a dissociation constant *K*_*D*_ = 0.01 nM (*k*_*on*_ = 0.1 L/nmol/min; *k*_*off*_ = 0.001 min^−1^): using 32 nmol compared to 1 nmol the normalized activity concentrations increased by a factor of 1.2 ± 0.1 for tumour lesions and 1.18 ± 0.03 for tumour REST (Supplement, Table S1). For the dissociation constant *K*_*D*_ = 1, the normalized activity concentrations were similar regardless of the administered ligand amount (1–32 nmol).

Organs at risk and background. Only for the dissociation constant *K*_*D*_ < 1 nM with an association rate *k*_*on*_ = 0.1 L/nmol/min the normalized activity concentrations varied considerably depending on the ligand amount. Otherwise the normalized activity concentrations in OARs and background were similar regardless of the ligand amounts (1–32 nmol) (Supplement, Tables S2 and S3).

#### Effect of the internalization rate *λ*_*int*_

Tumours, organs at risk and background. The parameter *λ*_*int*_ did not considerably affect the normalized activity concentration in tumour, all organs and background. The influence of the dissociation constant *K*_*D*_, association and dissociation rates *k*_*on*_ and *k*_*off*_ on the normalized activity concentration in tumour, OARs and background showed similar results for different internalization rates *λ*_*int*_ (Supplement, Tables S1–S3).

### Therapy

**Figure 2:**
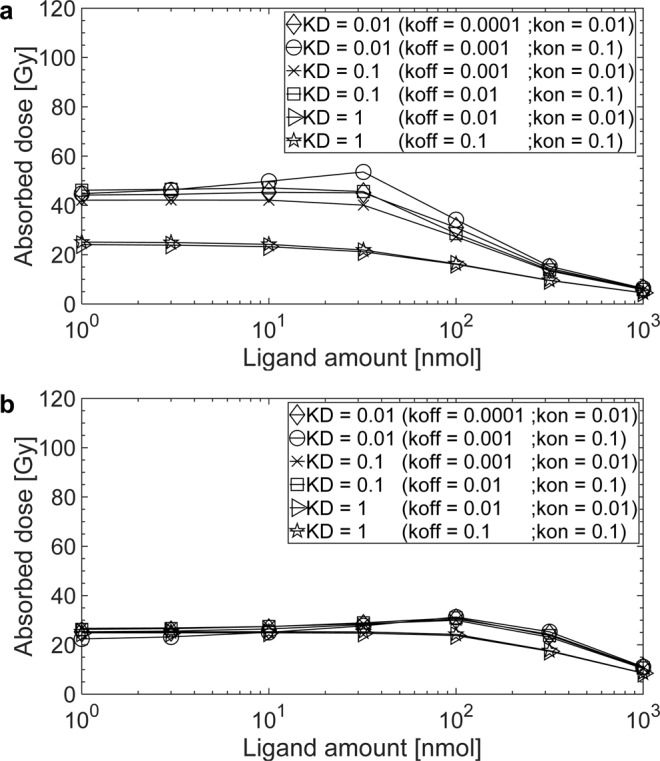
The absorbed doses for ^177^Lu-labelled (7.5 GBq) PSMA-specific ligands for the internalization rate λ_int_ = 0.001 min^−1^ in (**a**) a tumour lesion (receptor density 33 nmol/l and perfusion 0.08 ml/g/min) and (**b**) tumour REST (receptor density 66 nmol/l and perfusion 0.04 ml/g/min) of patient 5.

**Figure 3:**
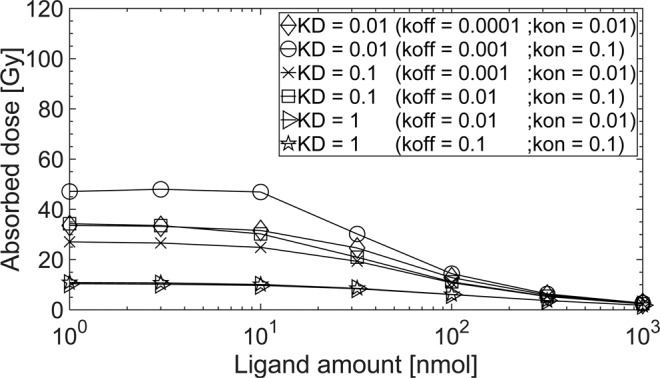
The absorbed dose for ^177^Lu-labelled (7.5 GBq) PSMA-specific ligands for the internalization rate λ_int_ = 0.001 min^−1^ in kidneys (receptor density 19 nmol/l and age adjusted perfusion 1.7 ml/g/min) of patient 5.

The results for therapy considering all patients, different ligand amounts, association and dissociation rates *k*_*on*_ and *k*_*off*_ and internalization rates *λ*_*int*_ are presented in the supplement (Table S5 for tumour, Table S6 for OARs and Table S7 for tumour-to-kidneys absorbed dose ratio). Figures 2a,b and 3 show the absorbed dose of the tumour lesions, the tumour REST and the kidneys depending on the association and dissociation rates *k*_*on*_ and *k*_*off*_ and on the applied ligand amount for a typical patient. The corresponding Figure for the red marrow is provided in the supplement (Fig. S2). All results described in the sections below refer to the internalization rate *λ*_*int*_ = 0.001 min^−1^, which is used in the published model^15^ and to ligand amounts relevant for therapy, i.e. 32–316 nmol.

#### Effect of association and dissociation rates *k*_*on*_ and *k*_*off*_

Tumours. Figure 2a,b show the dependence of the absorbed dose on the amount of ligand, association and dissociation rate *k*_*on*_ and *k*_*off*_, for tumour lesions and tumour REST, respectively. For the single tumour lesion with moderate blood flow and receptor density the effect of decreasing the dissociation constant *K*_*D*_ is considerable. The effect of different association and dissociation rates *k*_*on*_ and *k*_*off*_ for the same dissociation constant *K*_*D*_ is less prominent. Using a commonly applied ligand amount for therapy of 100 nmol, decreasing the dissociation constant *K*_*D*_ from 1 nM to 0.1 nM either by decreasing the dissociation rate *k*_*off*_ from 0.1 to 0.01 min^−1^ (with a fixed association rate *k*_*on*_ = 0.1 L/nmol/min) or by increasing association rate *k*_*on*_ from 0.01 to 0.1 min^−1^ (with a fixed dissociation rate *k*_*off*_ = 0.01 L/nmol/min) led to same results: the absorbed dose in tumour increased by a factor of 1.9 ± 0.2 for tumour lesions and 1.7 ± 0.4 for tumour REST (Supplement, Table S5).

For the tumour REST, which has a lower perfusion and a higher receptor density, association and dissociation rate *k*_*on*_ and *k*_*off*_ have only minimal influence for the investigated range. Figure 2a,b show that although the receptor density of tumour REST is two-fold larger, the two-fold lower perfusion diminishes the effect of higher affinity for the dissociation constant *K*_*D*_ < 1 nM.

Organs at risk. A decrease of the dissociation constant *K*_*D*_ resulted in an increase of the absorbed dose to other PSMA-positive organs in all patients (e.g. kidneys, Fig. 3). The absorbed doses increased by a factor of 2.1 ± 0.2 for kidneys when the dissociation constant *K*_*D*_ changed from 1 to 0.1 nM by decreasing the dissociation rate *k*_*off*_ from 0.1 to 0.01 min^−1^ (with a fixed association rate *k*_*on*_ = 0.1 L/nmol/min and a ligand amount of 100 nmol) (Supplement, Table S6). The absorbed doses in red marrow varied in the range of 0.06–0.23 Gy for all dissociation constant *K*_*D*_ values and ligand amounts in all patients (Supplement, Table S6).

#### Effect of ligand amount

Tumours. For all combinations of association and dissociation rate *k*_*on*_ and *k*_*off*_ the highest absorbed dose was simulated for 32 nmol. The decrease in the tumour absorbed dose with increasing ligand amount was more pronounced in highly perfused tissue for high affinities (Fig. 2a,b).

Organs at risk. The absorbed dose in the kidneys in general decreased with increasing ligand amount. For dissociation constant *K*_*D*_ < 1 nM with an association rate *k*_*on*_ = 0.1 L/nmol/min the absorbed dose in kidneys decreased stronger compared to larger dissociation constant *K*_*D*_ values. The ligand amount had a minor effect on the absorbed dose in red marrow.

#### Effect of the internalization rate *λ*_*int*_

Tumours. The effect of the internalization rate *λ*_*int*_ on the absorbed dose in the tumour varied considerably depending on the dissociation constant *K*_*D*_ and the ligand amount. For the dissociation constant *K*_*D*_ = 1 nM, the higher the internalization rate *λ*_*int*_ and ligand amount, the higher the absorbed dose in the tumour. The tumour absorbed dose increased by a factor of 2.0 ± 0.6 for the tumour lesions and 1.5 ± 0.6 for the tumour REST with an internalization rate *λ*_*int*_ = 0.01 min^−1^ compared to *λ*_*int*_ = 0.001 min^−1^ using a ligand amount of 32 nmol and the dissociation constant *K*_*D*_ = 1 nM (*k*_*off*_ = 0.01 min^−1^; *k*_*on*_ = 0.01 L/nmol/min). For affinities *K*_*D*_ < 1 nM and with an internalization rate *λ*_*in*_ = 0.01 min^−1^, a ligand amount > 32 nmol is required to achieve sufficient absorbed dose in the tumour. Conversely, for the internalization rate *λ*_*int*_ = 0.0001 min^−1^, a higher ligand amount led to a lower absorbed dose in the tumour (Supplement, Table S5).

Organs at risk. The absorbed dose in kidneys was higher for the internalization rate *λ*_*int*_ = 0.01 min^−1^ compared to *λ*_*int*_ = 0.001 min^−1^ for the all dissociation constant *K*_*D*_ using a ligand amount ≥ 32 nmol. The ligand amount affected the absorbed dose in the kidneys for the internalization rate *λ*_*int*_ = 0.01 min^−1^, however, only minor for the internalization rate *λ*_*int*_ = 0.0001 min^−1^. The absorbed dose in kidneys increased by a factor of 3.0 ± 0.3 with an internalization rate *λ*_*int*_ = 0.01 min^−1^ compared to the *λ*_*int*_ = 0.001 min^−1^ using ligand amount of 32 nmol and the dissociation constant *K*_*D*_ = 1 nM (*k*_*off*_ = 0.01 min^−1^; *k*_*on*_ = 0.01 L/nmol/min). The internalization rate *λ*_*int*_ only slightly affects the absorbed dose in red marrow regardless of the ligand amount (Supplement, Table S6).

#### Effects on tumour-to-kidneys absorbed dose ratio

The parameter values leading to the highest tumour-to-kidney absorbed dose ratio for different internalization rates *λ*_*int*_ (Table 2, Supplement, Table S7) were:

- For *λ*_*int*_ = 0.01 min^−1^: *k*_*on*_ = 0.01 L/nmol/min, *k*_*off*_ = 0.01 min^−1^ with ligand amount of 316 nmol (ratio of 3.0 ± 2.2 for tumour lesions and 3.0 ± 2.1 for tumour REST),
- For *λ*_*int*_ = 0.001 min^−1^: *k*_*on*_ = 0.1 L/nmol/min, *k*_*off*_ = 0.1 min^−1^ with ligand amount of 316 nmol (ratio of 3.8 ± 2.6 for tumour lesions and 4.9 ± 3.2 for tumour REST) and
- For *λ*_*int*_ = 0.0001 min^−1^: *k*_*on*_ = 0.1 L/nmol/min, *k*_*off*_ = 0.01 min^−1^ with ligand amount of 100 nmol (ratio of 3.8 ± 2.6 for tumour lesions and 5.1 ± 3.3 for tumour REST).

**Table 2:** The average of tumour-to-kidneys absorbed dose ratios considering different combinations *k*_*on*_ and *k*_*off*_, ligand amounts and *λ*_*int*_.

*K*_*D*_^*a*^ [nM]	*k*_*off*_^*b*^[1/ min]	*k*_*on*_^*c*^[L/ nmol/min]	Ligand amount [nmol]	Tumour-to-kidneys absorbed dose ratio of…					
				Tumour 1 and 2 for *λ*_*int*_^*d*^ of…			Tumour REST for *λ*_*int*_^*d*^ of…		
				0.01 [min^−1^]	0.001 [min^−1^]	0.0001 [min^−1^]	0.01 [min^−1^]	0.001 [min^−1^]	0.0001 [min^−1^]
0.01	0.0001	0.01	32	1.8 ± 1.3	2.3 ± 1.7	2.4 ± 1.7	1.3 ± 1.0	1.8 ± 1.4	2.2 ± 1.6
			100	2.2 ± 1.6	3.1 ± 2.3	3.1 ± 2.2	1.7 ± 1.4	3.1 ± 2.2	3.7 ± 2.6
			316	2.9 ± 2.1	3.5 ± 2.4	3.2 ± 2.1	2.7 ± 2.0	4.2 ± 2.8	4.2 ± 2.6
0.01	0.001	0.1	32	1.2 ± 0.9	2.2 ± 1.7	3.0 ± 2.3	0.8 ± 0.6	1.6 ± 1.4	2.7 ± 2.1
			100	1.8 ± 1.4	3.1 ± 2.3	3.4 ± 2.4	1.3 ± 1.1	3.1 ± 2.2	4.2 ± 3.0
			316	2.6 ± 1.9	3.4 ± 2.4	3.4 ± 2.3	2.3 ± 1.7	4.1 ± 2.8	4.6 ± 2.9
0.1	0.001	0.01	32	1.8 ± 1.3	2.6 ± 1.9	3.2 ± 2.3	1.4 ± 1.1	2.3 ± 1.7	3.6 ± 2.4
			100	2.2 ± 1.7	3.4 ± 2.5	3.5 ± 2.4	1.8 ± 1.4	3.5 ± 2.5	4.7 ± 3.1
			316	2.9 ± 2.1	3.6 ± 2.5	3.4 ± 2.3	2.7 ± 2.0	4.5 ± 3.0	4.8 ± 3.0
0.1	0.01	0.1	32	1.3 ± 1.0	2.8 ± 2.1	3.7 ± 2.6	0.9 ± 0.7	2.3 ± 1.8	4.2 ± 2.9
			100	1.9 ± 1.4	3.5 ± 2.6	**3.8** ± **2.6**	1.4 ± 1.2	3.7 ± 2.7	**5.1** ± **3.3**
			316	2.7 ± 1.9	3.7 ± 2.6	3.5 ± 2.4	2.4 ± 1.8	4.6 ± 3.1	5.0 ± 3.1
1	0.01	0.01	32	2.1 ± 1.6	3.4 ± 2.4	3.3 ± 2.2	1.7 ± 1.3	3.7 ± 2.5	4.3 ± 2.7
			100	2.5 ± 1.9	3.7 ± 2.6	3.3 ± 2.2	2.1 ± 1.6	4.4 ± 3.0	4.6 ± 2.9
			316	**3.0** ± **2.2**	3.7 ± 2.6	3.1 ± 2.0	**3.0** ± **2.1**	4.9 ± 3.2	4.4 ± 2.7
1	0.1	0.1	32	1.8 ± 1.4	3.5 ± 2.5	3.4 ± 2.3	1.4 ± 1.1	3.7 ± 2.6	4.4 ± 2.8
			100	2.3 ± 1.7	3.8 ± 2.7	3.3 ± 2.3	1.9 ± 1.5	4.5 ± 3.1	4.7 ± 2.9
			316	2.9 ± 2.1	**3.8** ± **2.6**	3.1 ± 2.0	2.8 ± 2.0	**4.9** ± **3.2**	4.4 ± 2.7

^*a*^*K*_*D*_ = Dissociation constant (*K*_*D*_ = *k*_*off*_/*k*_*on*_); ^*b*^*k*_*off*_ = Dissociation rate; ^*c*^*k*_*on*_ = Association rate; ^*d*^*λ*_*int*_ = Internalization rate. The values in bold are the maximum tumour-to-kidneys absorbed dose ratio.

## Discussion

Currently used ^68^Ga-labeled PSMA-specific ligands are effective in the detection of prostate cancer and ^177^Lu-labeled ligands show great potential in the treatment of metastatic prostate cancer^5^. For theranostic approaches, efforts are directed to use the same molecule for imaging and therapy with different labelling. This is challenging, as the ligands must be optimized for both at the same time, high tumour activity concentration with low background for imaging and a high tumour-to-OAR absorbed doses ratio. The influence of affinity, internalization and ligand amount on these quantities was investigated in this work based on a validated PBPK model^15^. The influence of the total tumour volume (tumour sink effect) and normal tissue uptake might be different for varying affinities and ligand amounts^15,22^. Different release rates (factor 2) and perfusion (factor 10) of kidneys and of tumour tissue might affect imaging and therapy differently.

A suitable method to systematically and quantitatively investigate these effects in various tissues concurrently is PBPK modelling^14,15^. A simulation study was therefore conducted using a recently developed PBPK model^15^ (based on data from PSMA-11 and PSMA I&T) for dissociation constant *K*_*D*_ values of 1, 0.1 and 0.01 nM (for each dissociation constant *K*_*D*_ two combinations of association and dissociation rates *k*_*on*_ and *k*_*off*_ were investigated), different internalization rates *λ*_*int*_ (0.01, 0.001 and 0.0001 min^−1^) and ligand amounts (1–1000 nmol). The simulation study yielded three major findings:

1. For imaging, ligands with *K*_*D*_ < 1 nM do not substantially increase the uptake in the tumour for commonly used ligand amounts compared to *K*_*D*_ = 1 nM as a) a considerable fraction is accumulated in highly perfused PSMA positive normal tissue and b) within one hour post injection differences might be considerably smaller compared to later time points. Therefore higher affinity might have a stronger effect on tumour activity concentration using nuclides with longer half-lives (^18^F or even ^64^Cu).
2. For therapy, a decreasing *K*_*D*_ considerably increases the absorbed dose in all PSMA positive tissue. The differences are the more prominent the higher the perfusion of the tissue and the lower the amount of ligand applied. Higher ligand amounts improve tumour-to-OAR absorbed dose ratios but result in lower absolute values. These results suggest that it is important to optimize the ligand amount and the pertaining activity to maximize efficacy within the dose limits of the OARs.
3. The simulations indicate that the combinations of association and dissociation rates *k*_*on*_ and *k*_*off*_ and ligand amount leading to the most favourable tumour-to-kidney ratios are:
  - For *λ*_*int*_ = 0.01 min^−1^: *k*_*on*_ = 0.01 L/nmol/min, *k*_*off*_ = 0.01 min^−1^ with ligand amount of 316 nmol
  - For *λ*_*in*__t _= 0.001 min^−1^: *k*_*on*_ = 0.1 L/nmol/min, *k*_*off*_ = 0.1 min^−1^ with ligand amount of 316 nmol
  - For *λ*_*int*_ = 0.0001 min^−1^: *k*_*on*_ = 0.1 L/nmol/min, *k*_*off*_ = 0.01 min^−1^ with ligand amount of 100 nmol

The optimal ligand amounts are a consequence of the more rapid saturation of highly perfused PSMA positive normal tissue where PSMA-specific binding is the dominant mechanism in our model.

In our model, all physiologically and physically relevant mechanisms such as perfusion, diffusion, internalization, serum protein binding, PSMA-specific binding are included. The PBPK structure is a trade-off between parsimony and biological reality. For some mechanisms and parameter values detailed knowledge is not available and thus was lumped or fitted. For example, the number of PSMA receptors (which were estimated in previous work using PSMA-11 and PSMA I&T data^15^) represent effective values including all receptor subtypes. In addition, complex internalization and recycling of the ligand and receptor was modelled with one single rate constant as reported for antibody pharmacokinetic modelling^18,19^. Although we have recently developed a more complex model for internalization of ^68^Ga-PSMA-11^21^, further experiments with different ligands and nuclides are required to integrate this model into the whole-body PBPK structure. The same holds true for salivary gland uptake. More experiments are required to identify the nature of the uptake mechanisms.

The unspecific uptake is assumed to be low given that the specific uptake affinity is high and amino acids were given to block unspecific uptake. For the kidneys, unspecific uptake is reported (and included in the model) but further research is needed to find the non-specific uptake mechanism^23^. If the unspecific uptake is larger than assumed (not blocked by amino acids and not saturable for high ligand amounts) the ligand amount leading to the optimal tumour-to-kidney absorbed dose ratio will tend to be smaller.

In the PBPK model it is assumed that the ligand which is transported over the capillary wall is instantaneously diffused and has access to binding sites. This is a good approximation for flow-limited small molecules as PSMA-11 and PSMA I&T and a maximal, diffusion-limited association rate *k*_*on*_ of 10^8^–10^9^ M^−1^·s^−17^. However, simply using association rate *k*_*on*_ from surface plasmon resonance measurements in this PBPK model might overestimate the actual binding because diffusion in the interstitial space is neglected.

In general, the translation of experimental values to *in vivo* systems is challenging. This is especially true for studies where *K*_*D*_ is derived at equilibrium whereas in the in vivo system with rapid clearance, binding equilibrium might not be reached. Our results nevertheless confirm that the separate experimental estimation of association and dissociation rates *k*_*on*_ and *k*_*off*_ and internalization rate *λ*_*int*_ is important^7^.

Incorporating further information of cell and animal experiments in this whole-body PBPK model is ongoing. In addition, for imaging the effect of using nuclides with a different half-life will be investigated.

## Conclusions

PBPK modelling proved to be a useful method for theoretically identifying ranges of ligand properties suitable for both imaging and therapy for theranostic applications. For the first time the interplay of important pharmacokinetic parameters for PSMA-specific ligands were investigated *in silico*. According to the simulations, association and dissociation rates that are optimal for therapy also lead to high tumour-to-background activity ratios for imaging. The results indicate that the properties of the ligands currently used are well chosen. Additionally, the simulations suggest that therapy might be considerably improved by choosing optimal activities *and* pertaining ligand amounts to achieve the highest tumour-to-OAR ratios. Further *in silico* and *in vivo* studies are required to verify the influence of the analysed parameters.

## Supplementary information

Supplementary results

## Data Availability

All data generated or analysed during this study are included in this article.
