# Supplementary material for: The effect of ligand amount, affinity and internalization on PSMA-targeted imaging and therapy: A simulation study using a PBPK model
Source: Sci Rep. 2019 Dec 27;9:20041. doi: 10.1038/s41598-019-56603-8 (PMC6934468; doi:10.1038/s41598-019-56603-8)
Supplement: Supplementary file 1 — Supplementary results [file 41598_2019_56603_MOESM1_ESM.docx]

**Supplementary results**

The effect of ligand amount, affinity and internalization on PSMA-targeted imaging and therapy: A simulation study using a PBPK model

Nusrat Jihan Begum^1*^, Gerhard Glatting^1,2^, Hans-Jürgen Wester^3^, Matthias Eiber^4^, Ambros J. Beer^2^, Peter Kletting^1,2^

*^1^Medical Radiation Physics, Department of Nuclear Medicine, Ulm University, Ulm, Germany*

*^2^Department of Nuclear Medicine, Ulm University, Ulm, Germany*

*^3^Technical University of Munich, Pharmaceutical Radiochemistry, Munich, Germany*

*^4^Technical University of Munich, School of Medicine, Klinikum rechts der Isar, Department of Nuclear Medicine, Munich, Germany*

^*^**Corresponding author:**

Nusrat Jihan Begum

Medizinische Strahlenphysik, Klinik für Nuklearmedizin, Universität Ulm

D - 89081 Ulm, Germany

Tel.: +49 731 500 61372

e-mail: [nusrat.begum@uni-ulm.de](mailto:nusrat.begum@uni-ulm.de)

| ***K_D_^a^***  **[nM]** | ***k_off_^b^***  **[1/min]** | ***k_on_^c^***  **[L/nmol/min]** | **Amount**  **[nmol]** | ***λ_int_^d^*=0.01 min^-1^** | | | ***λ_int_^d^*=0.001 min^-1^** | | | ***λ_int_^d^*=0.0001 min^-1^** | | |
| --- | --- | --- | --- | --- | --- | --- | --- | --- | --- | --- | --- | --- |
|  |  |  |  | **Activity concentration of…**  **[%Injected activity/l]** | | | **Activity concentration of…**  **[%Injected activity/l]** | | | **Activity concentration of…**  **[%Injected activity/l]** | | |
|  |  |  |  | **Tumour**  **1** | **Tumour**  **2** | **Tumour REST** | **Tumour**  **1** | **Tumour**  **2** | **Tumour REST** | **Tumour**  **1** | **Tumour**  **2** | **Tumour REST** |
| 0.01 | 0.0001 | 0.01 | 1 | 16±10 | 19±15 | 7±5 | 16±10 | 19±15 | 7±5 | 16±10 | 19±15 | 7±5 |
|  |  |  | 3 | 16±10 | 19±15 | 7±5 | 16±10 | 19±15 | 7±5 | 16±10 | 19±15 | 7±5 |
|  |  |  | 10 | 16±10 | 19±15 | 7±5 | 16±10 | 19±15 | 7±5 | 16±10 | 19±15 | 7±5 |
|  |  |  | 32 | 16±10 | 18±14 | 7±5 | 16±10 | 18±14 | 7±5 | 16±10 | 18±14 | 7±5 |
|  |  |  | 100 | 15±10 | 17±12 | 7±5 | 15±9 | 16±12 | 7±5 | 15±9 | 16±12 | 7±5 |
|  |  |  | 316 | 11±7 | 12±7 | 6±4 | 10±5 | 10±6 | 6±4 | 9±5 | 10±6 | 6±3 |
|  |  |  | 1000 | 5±3 | 5±3 | 4±2 | 4±2 | 4±2 | 3±2 | 4±2 | 4±2 | 3±2 |
| 0.01 | 0.001 | 0.1 | 1 | 16±10 | 20±18 | 7±5 | 16±10 | 20±18 | 7±5 | 16±10 | 20±18 | 7±5 |
|  |  |  | 3 | 16±11 | 21±18 | 7±6 | 17±11 | 21±18 | 7±6 | 17±11 | 21±18 | 7±6 |
|  |  |  | 10 | 17±11 | 22±19 | 7±6 | 18±11 | 22±19 | 8±6 | 18±11 | 22±20 | 8±6 |
|  |  |  | 32 | 19±12 | 23±21 | 8±6 | 19±12 | 23±21 | 8±6 | 19±12 | 23±21 | 8±6 |
|  |  |  | 100 | 19±12 | 22±19 | 8±6 | 19±13 | 20±15 | 8±6 | 18±13 | 20±14 | 8±6 |
|  |  |  | 316 | 13±8 | 13±9 | 7±4 | 10±6 | 10±6 | 6±4 | 10±5 | 10±6 | 6±4 |
|  |  |  | 1000 | 5±3 | 6±3 | 4±2 | 4±2 | 4±2 | 3±2 | 4±2 | 4±2 | 3±2 |
| 0.1 | 0.001 | 0.01 | 1 | 16±10 | 19±15 | 7±5 | 16±10 | 19±15 | 7±5 | 16±10 | 19±15 | 7±5 |
|  |  |  | 3 | 16±10 | 19±15 | 7±5 | 16±10 | 19±15 | 7±5 | 16±10 | 19±15 | 7±5 |
|  |  |  | 10 | 16±10 | 18±15 | 7±5 | 16±10 | 18±15 | 7±5 | 16±10 | 18±15 | 7±5 |
|  |  |  | 32 | 16±10 | 18±14 | 7±5 | 16±10 | 18±14 | 7±5 | 16±10 | 18±14 | 7±5 |
|  |  |  | 100 | 15±10 | 17±12 | 7±5 | 15±9 | 16±11 | 7±5 | 15±9 | 16±11 | 7±5 |
|  |  |  | 316 | 11±7 | 12±7 | 6±4 | 10±5 | 10±6 | 6±3 | 9±5 | 9±6 | 6±3 |
|  |  |  | 1000 | 5±3 | 5±3 | 4±2 | 4±2 | 4±2 | 3±2 | 4±2 | 4±2 | 3±2 |
| 0.1 | 0.01 | 0.1 | 1 | 16±11 | 21±18 | 7±5 | 17±11 | 21±18 | 7±6 | 17±11 | 21±18 | 7±6 |
|  |  |  | 3 | 17±11 | 21±18 | 7±6 | 17±11 | 21±19 | 7±6 | 17±11 | 21±19 | 7±6 |
|  |  |  | 10 | 18±11 | 22±19 | 8±6 | 18±11 | 22±19 | 8±6 | 18±11 | 22±19 | 8±6 |
|  |  |  | 32 | 19±12 | 23±20 | 8±6 | 19±12 | 23±20 | 8±6 | 19±12 | 23±20 | 8±6 |
|  |  |  | 100 | 19±12 | 22±18 | 8±6 | 18±12 | 20±15 | 8±6 | 18±12 | 19±14 | 8±6 |
|  |  |  | 316 | 13±8 | 13±8 | 7±4 | 10±6 | 10±6 | 6±4 | 10±5 | 10±6 | 6±4 |
|  |  |  | 1000 | 5±3 | 6±3 | 4±2 | 4±2 | 4±2 | 3±2 | 4±2 | 4±2 | 3±2 |
| 1 | 0.01 | 0.01 | 1 | 15±10 | 18±14 | 7±5 | 15±10 | 17±13 | 7±5 | 15±10 | 17±13 | 7±5 |
|  |  |  | 3 | 15±10 | 18±14 | 7±5 | 15±10 | 17±13 | 7±5 | 15±10 | 17±13 | 7±5 |
|  |  |  | 10 | 15±10 | 17±13 | 7±5 | 15±9 | 17±13 | 7±5 | 15±9 | 17±13 | 7±5 |
|  |  |  | 32 | 15±9 | 17±13 | 7±5 | 15±9 | 17±13 | 7±5 | 15±9 | 17±12 | 7±5 |
|  |  |  | 100 | 14±9 | 16±11 | 7±5 | 14±9 | 15±10 | 7±4 | 14±8 | 15±10 | 7±4 |
|  |  |  | 316 | 11±6 | 11±7 | 6±4 | 9±5 | 9±5 | 6±3 | 9±5 | 9±5 | 6±3 |
|  |  |  | 1000 | 5±2 | 5±3 | 4±2 | 4±2 | 4±2 | 3±2 | 4±2 | 4±2 | 3±2 |
| 1 | 0.1 | 0.1 | 1 | 17±11 | 21±18 | 8±6 | 17±11 | 21±18 | 8±6 | 17±11 | 21±17 | 8±6 |
|  |  |  | 3 | 17±11 | 21±18 | 8±6 | 17±11 | 21±18 | 8±6 | 17±11 | 21±17 | 8±6 |
|  |  |  | 10 | 18±11 | 21±18 | 8±6 | 17±11 | 21±17 | 8±6 | 17±11 | 21±17 | 8±6 |
|  |  |  | 32 | 18±11 | 21±18 | 8±6 | 17±11 | 20±17 | 8±6 | 17±11 | 20±17 | 8±6 |
|  |  |  | 100 | 17±11 | 19±15 | 8±6 | 16±10 | 17±12 | 8±5 | 16±10 | 17±12 | 7±5 |
|  |  |  | 316 | 12±7 | 12±8 | 7±4 | 9±5 | 9±5 | 6±4 | 9±5 | 9±5 | 6±4 |
|  |  |  | 1000 | 5±3 | 5±3 | 4±2 | 4±2 | 4±2 | 3±2 | 4±2 | 4±2 | 3±2 |

**Table S1.** The average of tumour activity concentrations of ^68^Ga-labeled PSMA-specific ligands (1 h p.i.) considering the different K_D_, k_on_, k_off_, λ_int_ and ligand amounts. ^a^K_D_=Dissociation constant (K_D_=k_off_/k_on_); ^b^k_off_ =Dissociation rate; ^c^k_on_=Association rate; ^d^λ_int_=Internalization rate.

| ***K_D_^a^***  **[nM]** | ***k_off_^b^***  **[1/min]** | ***k_on_^c^***  **[L/nmol/min]** | **Amount**  **[nmol]** | ***λ_int_^d^*=0.01 min^-1^** | | | ***λ_int_^d^*=0.001 min^-1^** | | | ***λ_int_^d^*=0.0001 min^-1^** | | |
| --- | --- | --- | --- | --- | --- | --- | --- | --- | --- | --- | --- | --- |
|  |  |  |  | **Activity concentration of…**  **[%Injected activity/l]** | | | **Activity concentration of…**  **[%Injected activity/l]** | | | **Activity concentration of…**  **[%Injected activity/l]** | | |
|  |  |  |  | **Kidneys** | **Liver** | **Red Marrow** | **Kidneys** | **Liver** | **Red Marrow** | **Kidneys** | **Liver** | **Red Marrow** |
| 0.01 | 0.0001 | 0.01 | 1 | 15±3 | 1.4±0.2 | 1.1±0.2 | 15±3 | 1.4±0.2 | 1.1±0.2 | 15±3 | 1.4±0.2 | 1.1±0.2 |
|  |  |  | 3 | 15±3 | 1.4±0.2 | 1.1±0.2 | 15±3 | 1.4±0.2 | 1.1±0.2 | 15±3 | 1.4±0.2 | 1.1±0.2 |
|  |  |  | 10 | 15±3 | 1.3±0.2 | 1.1±0.2 | 15±3 | 1.3±0.2 | 1.1±0.2 | 15±3 | 1.3±0.2 | 1.1±0.2 |
|  |  |  | 32 | 14±3 | 1.2±0.2 | 1.1±0.2 | 13±3 | 1.1±0.2 | 1.1±0.2 | 13±3 | 1.1±0.2 | 1.1±0.2 |
|  |  |  | 100 | 10±2 | 0.9±0.1 | 1.1±0.2 | 9±2 | 0.9±0.1 | 1.2±0.2 | 9±2 | 0.8±0.1 | 1.2±0.2 |
|  |  |  | 316 | 5±1 | 0.7±0.1 | 1.2±0.2 | 4±1 | 0.7±0.1 | 1.2±0.2 | 4±1 | 0.7±0.1 | 1.2±0.2 |
|  |  |  | 1000 | 2.6±0.4 | 0.7±0.1 | 1.2±0.2 | 2.2±0.3 | 0.6±0.1 | 1.2±0.2 | 2.2±0.3 | 0.6±0.1 | 1.2±0.2 |
| 0.01 | 0.001 | 0.1 | 1 | 24±5 | 6.2±0.7 | 0.7±0.1 | 24±5 | 6.1±0.7 | 0.8±0.1 | 24±5 | 6.1±0.7 | 0.8±0.1 |
|  |  |  | 3 | 25±6 | 5.5±0.6 | 0.8±0.1 | 25±6 | 5.2±0.5 | 0.8±0.1 | 25±6 | 5.2±0.5 | 0.8±0.1 |
|  |  |  | 10 | 26±6 | 3.7±0.3 | 0.9±0.2 | 26±6 | 3.2±0.2 | 0.9±0.2 | 26±6 | 3.1±0.2 | 1.0±0.2 |
|  |  |  | 32 | 26±6 | 1.8±0.1 | 1.0±0.2 | 25±5 | 1.5±0.1 | 1.1±0.2 | 25±5 | 1.4±0.1 | 1.1±0.2 |
|  |  |  | 100 | 15±2 | 1.0±0.1 | 1.1±0.2 | 11±2 | 0.9±0.1 | 1.1±0.2 | 11±2 | 0.9±0.1 | 1.1±0.2 |
|  |  |  | 316 | 6±1 | 0.7±0.1 | 1.2±0.2 | 4±1 | 0.7±0.1 | 1.2±0.2 | 4±1 | 0.7±0.1 | 1.2±0.2 |
|  |  |  | 1000 | 2.7±0.4 | 0.7±0.1 | 1.2±0.2 | 2.2±0.3 | 0.6±0.1 | 1.2±0.2 | 2.1±0.3 | 0.6±0.1 | 1.2±0.2 |
| 0.1 | 0.001 | 0.01 | 1 | 15±3 | 1.4±0.2 | 1.1±0.2 | 15±3 | 1.4±0.2 | 1.1±0.2 | 15±3 | 1.4±0.2 | 1.1±0.2 |
|  |  |  | 3 | 15±3 | 1.4±0.2 | 1.1±0.2 | 15±3 | 1.4±0.2 | 1.1±0.2 | 15±3 | 1.4±0.2 | 1.1±0.2 |
|  |  |  | 10 | 15±3 | 1.3±0.2 | 1.1±0.2 | 15±3 | 1.3±0.2 | 1.1±0.2 | 15±3 | 1.3±0.2 | 1.1±0.2 |
|  |  |  | 32 | 14±3 | 1.2±0.2 | 1.1±0.2 | 13±3 | 1.1±0.2 | 1.1±0.2 | 13±3 | 1.1±0.2 | 1.1±0.2 |
|  |  |  | 100 | 10±2 | 0.9±0.1 | 1.1±0.2 | 9±2 | 0.9±0.1 | 1.2±0.2 | 9±2 | 0.8±0.1 | 1.2±0.2 |
|  |  |  | 316 | 5±1 | 0.7±0.1 | 1.2±0.2 | 4±1 | 0.7±0.1 | 1.2±0.2 | 4±1 | 0.7±0.1 | 1.2±0.2 |
|  |  |  | 1000 | 2.6±0.4 | 0.7±0.1 | 1.2±0.2 | 2.2±0.3 | 0.6±0.1 | 1.2±0.2 | 2.1±0.3 | 0.6±0.1 | 1.2±0.2 |
| 0.1 | 0.01 | 0.1 | 1 | 24±5 | 5.2±0.6 | 0.8±0.1 | 24±5 | 4.9±0.6 | 0.8±0.1 | 24±5 | 4.9±0.6 | 0.8±0.1 |
|  |  |  | 3 | 25±5 | 4.7±0.5 | 0.9±0.1 | 25±5 | 4.3±0.5 | 0.9±0.1 | 25±5 | 4.2±0.5 | 0.9±0.1 |
|  |  |  | 10 | 26±6 | 3.3±0.3 | 0.9±0.2 | 26±6 | 2.8±0.2 | 1.0±0.2 | 26±6 | 2.7±0.2 | 1.0±0.2 |
|  |  |  | 32 | 25±5 | 1.7±0.1 | 1.1±0.2 | 24±5 | 1.4±0.1 | 1.1±0.2 | 23±5 | 1.4±0.1 | 1.1±0.2 |
|  |  |  | 100 | 14±2 | 1.0±0.1 | 1.1±0.2 | 11±2 | 0.9±0.1 | 1.1±0.2 | 10±2 | 0.8±0.1 | 1.1±0.2 |
|  |  |  | 316 | 6±1 | 0.7±0.1 | 1.2±0.2 | 4±1 | 0.7±0.1 | 1.2±0.2 | 4±1 | 0.7±0.1 | 1.2±0.2 |
|  |  |  | 1000 | 2.7±0.4 | 0.7±0.1 | 1.2±0.2 | 2.2±0.3 | 0.6±0.1 | 1.2±0.2 | 2.1±0.3 | 0.6±0.1 | 1.2±0.2 |
| 1 | 0.01 | 0.01 | 1 | 13±3 | 1.2±0.2 | 1.1±0.2 | 13±3 | 1.2±0.2 | 1.1±0.2 | 13±3 | 1.2±0.2 | 1.1±0.2 |
|  |  |  | 3 | 13±3 | 1.2±0.2 | 1.1±0.2 | 13±3 | 1.2±0.2 | 1.1±0.2 | 13±3 | 1.2±0.2 | 1.1±0.2 |
|  |  |  | 10 | 13±3 | 1.2±0.2 | 1.1±0.2 | 12±3 | 1.1±0.2 | 1.1±0.2 | 12±3 | 1.1±0.2 | 1.1±0.2 |
|  |  |  | 32 | 12±3 | 1.1±0.2 | 1.1±0.2 | 11±2 | 1.0±0.2 | 1.1±0.2 | 11±2 | 1.0±0.2 | 1.1±0.2 |
|  |  |  | 100 | 9±2 | 0.9±0.1 | 1.2±0.2 | 8±2 | 0.8±0.1 | 1.2±0.2 | 8±1 | 0.8±0.1 | 1.2±0.2 |
|  |  |  | 316 | 5±1 | 0.7±0.1 | 1.2±0.2 | 4±1 | 0.7±0.1 | 1.2±0.2 | 4±1 | 0.7±0.1 | 1.2±0.2 |
|  |  |  | 1000 | 2.6±0.4 | 0.7±0.1 | 1.2±0.2 | 2.2±0.3 | 0.6±0.1 | 1.3±0.2 | 2.1±0.3 | 0.6±0.1 | 1.3±0.2 |
| 1 | 0.1 | 0.1 | 1 | 21±4 | 2.1±0.3 | 1.0±0.2 | 20±4 | 1.7±0.3 | 1.1±0.2 | 20±4 | 1.6±0.3 | 1.1±0.2 |
|  |  |  | 3 | 21±4 | 2.0±0.3 | 1.1±0.2 | 20±4 | 1.6±0.3 | 1.1±0.2 | 20±4 | 1.5±0.3 | 1.1±0.2 |
|  |  |  | 10 | 20±4 | 1.7±0.2 | 1.1±0.2 | 19±4 | 1.4±0.2 | 1.1±0.2 | 19±4 | 1.3±0.2 | 1.1±0.2 |
|  |  |  | 32 | 18±4 | 1.3±0.2 | 1.1±0.2 | 16±3 | 1.1±0.2 | 1.1±0.2 | 15±3 | 1.1±0.2 | 1.1±0.2 |
|  |  |  | 100 | 12±2 | 0.9±0.1 | 1.1±0.2 | 9±2 | 0.8±0.1 | 1.1±0.2 | 8±2 | 0.8±0.1 | 1.2±0.2 |
|  |  |  | 316 | 5±1 | 0.7±0.1 | 1.2±0.2 | 4±1 | 0.7±0.1 | 1.2±0.2 | 4±1 | 0.7±0.1 | 1.2±0.2 |
|  |  |  | 1000 | 2.6±0.4 | 0.7±0.1 | 1.2±0.2 | 2.2±0.3 | 0.6±0.1 | 1.2±0.2 | 2.1±0.3 | 0.6±0.1 | 1.2±0.2 |

**Table S2.** The average of kidneys, liver and red marrow activity concentrations of ^68^Ga-labeled PSMA-specific ligands (1 h p.i.) considering the different K_D_, k_on_, k_off_, λ_int_ and ligand amounts. ^a^K_D_=Dissociation constant (K_D_=k_off_/k_on_); ^b^k_off_ =Dissociation rate; ^c^k_on_=Association rate; ^d^λ_int_=Internalization rate.

| ***K_D_^a^***  **[nM]** | ***k_off_^b^***  **[1/min]** | ***k_on_^c^***  **[L/nmol/min]** | **Amount**  **[nmol]** | ***λ_int_^d^*=0.01 min^-1^** | | | ***λ_int_^d^*=0.001 min^-1^** | | | ***λ_int_^d^*=0.0001 min^-1^** | | |
| --- | --- | --- | --- | --- | --- | --- | --- | --- | --- | --- | --- | --- |
|  |  |  |  | **Activity concentration of…**  **[%Injected activity/l]** | | | **Activity concentration of…**  **[%Injected activity/l]** | | | **Activity concentration of…**  **[%Injected activity/l]** | | |
|  |  |  |  | **Background** | **GI** | **Lung** | **Background** | **GI** | **Lung** | **Background** | **GI** | **Lung** |
| 0.01 | 0.0001 | 0.01 | 1 | 0.28±0.04 | 2.8±0.4 | 4±1 | 0.28±0.04 | 2.8±0.4 | 4±1 | 0.28±0.04 | 2.8±0.4 | 4±1 |
|  |  |  | 3 | 0.28±0.04 | 2.8±0.4 | 4±1 | 0.28±0.04 | 2.8±0.4 | 4±1 | 0.28±0.04 | 2.8±0.4 | 4±1 |
|  |  |  | 10 | 0.28±0.04 | 2.8±0.4 | 4±1 | 0.28±0.04 | 2.8±0.4 | 4±1 | 0.28±0.04 | 2.8±0.4 | 4±1 |
|  |  |  | 32 | 0.28±0.04 | 2.8±0.4 | 4±1 | 0.28±0.04 | 2.8±0.4 | 4±1 | 0.28±0.04 | 2.8±0.4 | 4±1 |
|  |  |  | 100 | 0.29±0.05 | 2.8±0.4 | 4±1 | 0.29±0.05 | 2.8±0.4 | 4±1 | 0.29±0.05 | 2.8±0.4 | 4±1 |
|  |  |  | 316 | 0.30±0.05 | 2.9±0.4 | 5±1 | 0.30±0.05 | 2.9±0.4 | 5±1 | 0.30±0.05 | 2.9±0.4 | 5±1 |
|  |  |  | 1000 | 0.31±0.05 | 2.9±0.4 | 5±1 | 0.31±0.05 | 2.9±0.4 | 5±1 | 0.31±0.05 | 2.9±0.4 | 5±1 |
| 0.01 | 0.001 | 0.1 | 1 | 0.20±0.03 | 2.5±0.3 | 2.8±0.4 | 0.20±0.03 | 2.5±0.3 | 2.8±0.4 | 0.20±0.03 | 2.5±0.3 | 2.8±0.4 |
|  |  |  | 3 | 0.21±0.03 | 2.6±0.3 | 3.0±0.5 | 0.21±0.03 | 2.6±0.3 | 3.0±0.5 | 0.21±0.03 | 2.6±0.3 | 3±1 |
|  |  |  | 10 | 0.23±0.04 | 2.7±0.4 | 3±1 | 0.24±0.04 | 2.8±0.4 | 4±1 | 0.24±0.04 | 2.8±0.4 | 4±1 |
|  |  |  | 32 | 0.26±0.04 | 2.9±0.4 | 4±1 | 0.27±0.04 | 2.9±0.4 | 4±1 | 0.27±0.04 | 2.9±0.4 | 4±1 |
|  |  |  | 100 | 0.28±0.05 | 2.9±0.4 | 4±1 | 0.28±0.05 | 2.9±0.4 | 4±1 | 0.28±0.05 | 2.9±0.4 | 4±1 |
|  |  |  | 316 | 0.29±0.05 | 2.9±0.4 | 4±1 | 0.30±0.05 | 2.9±0.4 | 5±1 | 0.30±0.05 | 2.9±0.4 | 5±1 |
|  |  |  | 1000 | 0.30±0.05 | 2.9±0.4 | 5±1 | 0.31±0.05 | 2.9±0.4 | 5±1 | 0.31±0.05 | 2.9±0.4 | 5±1 |
| 0.1 | 0.001 | 0.01 | 1 | 0.28±0.04 | 2.8±0.4 | 4±1 | 0.28±0.04 | 2.8±0.4 | 4±1 | 0.28±0.04 | 2.8±0.4 | 4±1 |
|  |  |  | 3 | 0.28±0.04 | 2.8±0.4 | 4±1 | 0.28±0.04 | 2.8±0.4 | 4±1 | 0.28±0.04 | 2.8±0.4 | 4±1 |
|  |  |  | 10 | 0.28±0.04 | 2.8±0.4 | 4±1 | 0.28±0.04 | 2.8±0.4 | 4±1 | 0.28±0.04 | 2.8±0.4 | 4±1 |
|  |  |  | 32 | 0.28±0.04 | 2.8±0.4 | 4±1 | 0.28±0.04 | 2.8±0.4 | 4±1 | 0.28±0.04 | 2.8±0.4 | 4±1 |
|  |  |  | 100 | 0.29±0.05 | 2.8±0.4 | 4±1 | 0.29±0.05 | 2.8±0.4 | 4±1 | 0.29±0.05 | 2.8±0.4 | 4±1 |
|  |  |  | 316 | 0.30±0.05 | 2.9±0.4 | 5±1 | 0.30±0.05 | 2.9±0.4 | 5±1 | 0.30±0.05 | 2.9±0.4 | 5±1 |
|  |  |  | 1000 | 0.31±0.05 | 2.9±0.4 | 5±1 | 0.31±0.05 | 2.9±0.4 | 5±1 | 0.31±0.05 | 2.9±0.4 | 5±1 |
| 0.1 | 0.01 | 0.1 | 1 | 0.21±0.03 | 2.6±0.3 | 3.1±0.5 | 0.22±0.03 | 2.6±0.3 | 3.2±0.5 | 0.22±0.03 | 2.6±0.3 | 3.2±0.5 |
|  |  |  | 3 | 0.22±0.03 | 2.6±0.4 | 3±1 | 0.23±0.03 | 2.6±0.4 | 3±1 | 0.23±0.03 | 2.6±0.4 | 3±1 |
|  |  |  | 10 | 0.24±0.04 | 2.7±0.4 | 4±1 | 0.25±0.04 | 2.8±0.4 | 4±1 | 0.25±0.04 | 2.8±0.4 | 4±1 |
|  |  |  | 32 | 0.27±0.04 | 2.9±0.4 | 4±1 | 0.27±0.04 | 2.9±0.4 | 4±1 | 0.27±0.04 | 2.9±0.4 | 4±1 |
|  |  |  | 100 | 0.28±0.05 | 2.9±0.4 | 4±1 | 0.28±0.05 | 2.9±0.4 | 4±1 | 0.28±0.05 | 2.9±0.4 | 4±1 |
|  |  |  | 316 | 0.29±0.05 | 2.9±0.4 | 4±1 | 0.30±0.05 | 2.9±0.4 | 5±1 | 0.30±0.05 | 2.9±0.4 | 5±1 |
|  |  |  | 1000 | 0.30±0.05 | 2.9±0.4 | 5±1 | 0.31±0.05 | 2.9±0.4 | 5±1 | 0.31±0.05 | 2.9±0.4 | 5±1 |
| 1 | 0.01 | 0.01 | 1 | 0.28±0.04 | 2.8±0.4 | 4±1 | 0.28±0.04 | 2.8±0.4 | 4±1 | 0.28±0.04 | 2.8±0.4 | 4±1 |
|  |  |  | 3 | 0.28±0.04 | 2.8±0.4 | 4±1 | 0.28±0.04 | 2.8±0.4 | 4±1 | 0.28±0.04 | 2.8±0.4 | 4±1 |
|  |  |  | 10 | 0.28±0.04 | 2.8±0.4 | 4±1 | 0.28±0.04 | 2.8±0.4 | 4±1 | 0.28±0.04 | 2.8±0.4 | 4±1 |
|  |  |  | 32 | 0.29±0.04 | 2.8±0.4 | 4±1 | 0.29±0.04 | 2.8±0.4 | 4±1 | 0.29±0.04 | 2.8±0.4 | 4±1 |
|  |  |  | 100 | 0.29±0.05 | 2.8±0.4 | 4±1 | 0.29±0.05 | 2.8±0.4 | 4±1 | 0.29±0.05 | 2.8±0.4 | 4±1 |
|  |  |  | 316 | 0.30±0.05 | 2.9±0.4 | 5±1 | 0.30±0.05 | 2.9±0.4 | 5±1 | 0.30±0.05 | 2.9±0.4 | 5±1 |
|  |  |  | 1000 | 0.31±0.05 | 2.9±0.4 | 5±1 | 0.31±0.05 | 2.9±0.4 | 5±1 | 0.31±0.05 | 2.9±0.4 | 5±1 |
| 1 | 0.1 | 0.1 | 1 | 0.26±0.04 | 2.7±0.4 | 4±1 | 0.27±0.04 | 2.8±0.4 | 4±1 | 0.27±0.04 | 2.8±0.4 | 4±1 |
|  |  |  | 3 | 0.27±0.04 | 2.8±0.4 | 4±1 | 0.27±0.04 | 2.8±0.4 | 4±1 | 0.27±0.04 | 2.8±0.4 | 4±1 |
|  |  |  | 10 | 0.27±0.04 | 2.8±0.4 | 4±1 | 0.28±0.04 | 2.8±0.4 | 4±1 | 0.28±0.04 | 2.8±0.4 | 4±1 |
|  |  |  | 32 | 0.28±0.04 | 2.8±0.4 | 4±1 | 0.28±0.04 | 2.8±0.4 | 4±1 | 0.28±0.04 | 2.8±0.4 | 4±1 |
|  |  |  | 100 | 0.28±0.05 | 2.8±0.4 | 4±1 | 0.29±0.05 | 2.8±0.4 | 4±1 | 0.29±0.05 | 2.8±0.4 | 4±1 |
|  |  |  | 316 | 0.29±0.05 | 2.9±0.4 | 5±1 | 0.30±0.05 | 2.9±0.4 | 5±1 | 0.30±0.05 | 2.9±0.4 | 5±1 |
|  |  |  | 1000 | 0.31±0.05 | 2.9±0.4 | 5±1 | 0.31±0.05 | 2.9±0.4 | 5±1 | 0.31±0.05 | 2.9±0.4 | 5±1 |

**Table S3.** The average of background, gastro intestinal (GI) and lung activity concentrations of ^68^Ga-labeled PSMA-specific ligands (1 h p.i.) considering the different K_D_, k_on_, k_off_, λ_int_ and ligand amounts. ^a^K_D_=Dissociation constant (K_D_=k_off_/k_on_); ^b^k_off_ =Dissociation rate; ^c^k_on_=Association rate; ^d^λ_int_=Internalization rate.

| **Decreasing *K_D_^a^* from…**  **[nM]** | ***k_off_^b^***  [min^-1^] | ***k_on_^c^***  **[L/nmol/min]** | **Amount**  **[nmol]** | **The ratio of the normalized activity concentrations of…** | |
| --- | --- | --- | --- | --- | --- |
|  |  |  |  | **Tumour 1 and 2** | **Tumour REST** |
| 1 to 0.1 | 0.1 to 0.01 | fixed  (0.1) | 3 | 1.0±0.1 | 0.94±0.04 |
|  |  |  | 10 | 1.0±0.1 | 0.98±0.04 |
|  |  |  | 32 | 1.1±0.1 | 1.0±0.1 |
| 1 to 0.01 | 0.1 to 0.001 | fixed  (0.1) | 3 | 1.0±0.1 | 0.92±0.04 |
|  |  |  | 10 | 1.0±0.1 | 0.97±0.05 |
|  |  |  | 32 | 1.1±0.1 | 1.0±0.1 |
| 1 to 0.1 | 0.01 to 0.001 | fixed  (0.01) | 3 | 1.06±0.03 | 1.03±0.03 |
|  |  |  | 10 | 1.06±0.03 | 1.03±0.03 |
|  |  |  | 32 | 1.06±0.04 | 1.03±0.03 |
| 1 to 0.01 | 0.01 to 0.0001 | fixed  (0.01) | 3 | 1.06±0.04 | 1.03±0.03 |
|  |  |  | 10 | 1.07±0.04 | 1.03±0.03 |
|  |  |  | 32 | 1.07±0.04 | 1.04±0.03 |
| 1 to 0.1 | fixed  (0.01) | 0.01 to 0.1 | 3 | 1.2±0.3 | 1.0±0.1 |
|  |  |  | 10 | 1.2±0.3 | 1.1±0.1 |
|  |  |  | 32 | 1.3±0.2 | 1.1±0.2 |
| 0.1 to 0.01 | fixed  (0.001) | 0.01 to 0.1 | 3 | 1.1±0.2 | 1.0±0.1 |
|  |  |  | 10 | 1.2±0.3 | 1.0±0.1 |
|  |  |  | 32 | 1.2±0.2 | 1.1±0.1 |

**Table S4.** The ratio of the normalized activity concentrations in tumour by decreasing K_D_ by changing k_off_ or k_on_. ^a^K_D_=Dissociation constant (K_D_=k_off_/k_on_); ^b^k_off_ =Dissociation rate; ^c^k_on_=Association rate.

**Figure S1.** Normalized activity concentrations for ^68^Ga-labeled (1 h p.i.) PSMA-specific ligands for background (muscle and fat) of patient 5.

| ***K_D_^a^***  **[nM]** | ***k_off_^b^***  **[1/min]** | ***k_on_^c^***  **[L/nmol/min]** | **Amount**  **[nmol]** | ***λ_int_^d^*=0.01 min^-1^** | | | ***λ_int_^d^*=0.001 min^-1^** | | | ***λ_int_^d^*=0.0001 min^-1^** | | |
| --- | --- | --- | --- | --- | --- | --- | --- | --- | --- | --- | --- | --- |
|  |  |  |  | **Absorbed dose of…**  **[Gy]** | | | **Absorbed dose of…**  **[Gy]** | | | **Absorbed dose of…**  **[Gy]** | | |
|  |  |  |  | **Tumour**  **1** | **Tumour**  **2** | **Tumour REST** | **Tumour**  **1** | **Tumour**  **2** | **Tumour REST** | **Tumour**  **1** | **Tumour**  **2** | **Tumour REST** |
| 0.01 | 0.0001 | 0.01 | 1 | 37±20 | 44±32 | 29±20 | 42±23 | 50±35 | 34±23 | 65±37 | 74±52 | 55±37 |
|  |  |  | 3 | 37±20 | 44±32 | 29±20 | 43±23 | 50±35 | 34±23 | 65±37 | 74±51 | 55±37 |
|  |  |  | 10 | 38±20 | 44±32 | 30±20 | 43±23 | 50±35 | 35±23 | 64±37 | 72±49 | 55±37 |
|  |  |  | 32 | 38±21 | 45±32 | 30±20 | 42±24 | 47±32 | 35±22 | 57±35 | 61±40 | 54±33 |
|  |  |  | 100 | 38±21 | 43±30 | 31±20 | 31±19 | 33±23 | 32±19 | 33±21 | 34±23 | 40±24 |
|  |  |  | 316 | 30±18 | 33±22 | 29±17 | 15±10 | 16±11 | 19±12 | 13±8 | 13±9 | 17±10 |
|  |  |  | 1000 | 17±11 | 19±13 | 20±13 | 6±4 | 7±5 | 8±5 | 4±3 | 5±3 | 6±3 |
| 0.01 | 0.001 | 0.1 | 1 | 30±16 | 38±30 | 22±17 | 45±23 | 55±42 | 33±25 | 75±42 | 89±67 | 60±45 |
|  |  |  | 3 | 31±17 | 39±31 | 23±18 | 46±24 | 56±43 | 34±25 | 76±42 | 89±66 | 61±45 |
|  |  |  | 10 | 34±18 | 43±33 | 25±19 | 49±25 | 59±45 | 37±27 | 75±43 | 85±60 | 61±44 |
|  |  |  | 32 | 39±20 | 48±38 | 29±22 | 49±28 | 56±40 | 39±27 | 63±41 | 64±43 | 58±36 |
|  |  |  | 100 | 43±23 | 50±37 | 33±23 | 34±22 | 37±26 | 35±20 | 32±21 | 34±22 | 40±25 |
|  |  |  | 316 | 34±20 | 38±26 | 32±19 | 16±10 | 17±12 | 20±13 | 12±8 | 13±8 | 17±10 |
|  |  |  | 1000 | 19±12 | 21±14 | 22±13 | 6±4 | 7±5 | 8±5 | 4±3 | 5±3 | 6±3 |
| 0.1 | 0.001 | 0.01 | 1 | 37±20 | 44±32 | 29±20 | 40±22 | 45±31 | 33±21 | 41±25 | 44±27 | 41±24 |
|  |  |  | 3 | 37±20 | 44±32 | 29±20 | 40±22 | 45±31 | 33±21 | 41±24 | 44±27 | 41±24 |
|  |  |  | 10 | 38±20 | 44±32 | 30±20 | 39±22 | 44±30 | 33±21 | 39±24 | 41±25 | 40±23 |
|  |  |  | 32 | 38±21 | 44±31 | 30±20 | 37±22 | 41±27 | 33±20 | 33±21 | 34±21 | 37±21 |
|  |  |  | 100 | 37±21 | 42±29 | 31±20 | 27±17 | 29±20 | 29±17 | 19±13 | 20±14 | 26±16 |
|  |  |  | 316 | 30±18 | 32±22 | 29±17 | 14±9 | 15±10 | 17±11 | 8±5 | 9±6 | 12±7 |
|  |  |  | 1000 | 17±11 | 19±13 | 20±12 | 6±4 | 6±4 | 8±5 | 3±2 | 3±2 | 4±3 |
| 0.1 | 0.01 | 0.1 | 1 | 33±18 | 41±32 | 25±19 | 44±24 | 51±37 | 35±24 | 44±26 | 47±29 | 43±25 |
|  |  |  | 3 | 34±18 | 42±33 | 25±19 | 44±24 | 51±36 | 35±24 | 43±26 | 46±28 | 43±25 |
|  |  |  | 10 | 36±19 | 45±35 | 27±20 | 44±24 | 51±36 | 36±24 | 41±25 | 43±26 | 42±24 |
|  |  |  | 32 | 40±21 | 49±37 | 30±22 | 42±24 | 45±31 | 36±22 | 33±21 | 33±22 | 38±22 |
|  |  |  | 100 | 43±23 | 49±36 | 33±23 | 29±19 | 31±21 | 31±19 | 19±13 | 19±14 | 26±17 |
|  |  |  | 316 | 34±20 | 37±26 | 31±19 | 14±9 | 15±10 | 18±12 | 8±5 | 8±6 | 12±7 |
|  |  |  | 1000 | 19±12 | 21±14 | 21±13 | 6±4 | 6±4 | 8±5 | 3±2 | 3±2 | 4±3 |
| 1 | 0.01 | 0.01 | 1 | 35±19 | 41±28 | 29±19 | 22±14 | 24±15 | 23±13 | 10±6 | 11±7 | 13±7 |
|  |  |  | 3 | 35±19 | 41±28 | 29±19 | 22±13 | 23±15 | 23±13 | 10±6 | 11±7 | 13±7 |
|  |  |  | 10 | 35±19 | 40±28 | 29±19 | 21±13 | 23±15 | 22±13 | 10±6 | 10±6 | 13±7 |
|  |  |  | 32 | 35±19 | 40±27 | 29±18 | 20±12 | 21±14 | 22±13 | 9±6 | 9±6 | 11±7 |
|  |  |  | 100 | 33±19 | 37±25 | 29±18 | 15±10 | 16±11 | 19±12 | 6±4 | 7±4 | 9±5 |
|  |  |  | 316 | 27±16 | 29±20 | 27±16 | 9±6 | 10±7 | 12±8 | 3±2 | 4±2 | 5±3 |
|  |  |  | 1000 | 16±11 | 18±12 | 19±12 | 4±3 | 5±3 | 6±4 | 2±1 | 2±1 | 2±1 |
| 1 | 0.1 | 0.1 | 1 | 37±20 | 44±32 | 30±20 | 23±14 | 25±16 | 24±14 | 10±6 | 11±7 | 13±7 |
|  |  |  | 3 | 37±20 | 44±32 | 30±20 | 23±14 | 25±16 | 24±14 | 10±6 | 11±7 | 13±7 |
|  |  |  | 10 | 38±20 | 44±32 | 30±20 | 22±14 | 24±15 | 23±14 | 10±6 | 10±6 | 13±7 |
|  |  |  | 32 | 38±21 | 44±32 | 31±20 | 20±13 | 22±14 | 22±13 | 9±6 | 9±6 | 11±7 |
|  |  |  | 100 | 37±21 | 42±29 | 31±19 | 15±10 | 16±11 | 19±12 | 6±4 | 6±4 | 9±5 |
|  |  |  | 316 | 29±18 | 32±22 | 29±17 | 9±6 | 10±7 | 12±8 | 3±2 | 4±2 | 5±3 |
|  |  |  | 1000 | 17±11 | 19±13 | 20±12 | 4±3 | 5±3 | 6±4 | 1±1 | 2±1 | 2±1 |

**Table S5**. The average of tumour absorbed doses of ^177^Lu-labeled PSMA-specific ligands considering the different K_D_, k_off_, k_on_, λ_int_ and ligand amounts. ^a^K_D_=Dissociation constant (K_D_=k_off_/k_on_); ^b^k_off_ =Dissociation rate; ^c^k_on_=Association rate; ^d^λ_int_=Internalization rate.

| ***K_D_^a^***  **[nM]** | ***k_off_^b^***  **[1/min]** | ***k_on_^c^***  **[L/nmol/min]** | **Amount**  **[nmol]** | ***λ_int_^d^*=0.01 min^-1^** | | ***λ_int_^d^*=0.001 min^-1^** | | ***λ_int_^d^*=0.0001 min^-1^** | |
| --- | --- | --- | --- | --- | --- | --- | --- | --- | --- |
|  |  |  |  | **Absorbed dose of…**  **[Gy]** | | **Absorbed dose of…**  **[Gy]** | | **Absorbed dose of…**  **[Gy]** | |
|  |  |  |  | **Kidneys** | **Red Marrow** | **Kidneys** | **Red Marrow** | **Kidneys** | **Red Marrow** |
| 0.01 | 0.0001 | 0.01 | 1 | 28±7 | 0.13±0.04 | 31±8 | 0.14±0.05 | 48±14 | 0.19±0.06 |
|  |  |  | 3 | 28±7 | 0.13±0.04 | 30±8 | 0.14±0.05 | 46±14 | 0.19±0.06 |
|  |  |  | 10 | 27±7 | 0.13±0.04 | 28±7 | 0.14±0.05 | 41±11 | 0.18±0.06 |
|  |  |  | 32 | 26±7 | 0.13±0.04 | 21±5 | 0.14±0.05 | 26±5 | 0.17±0.07 |
|  |  |  | 100 | 20±6 | 0.13±0.05 | 11±2 | 0.13±0.05 | 11±2 | 0.15±0.07 |
|  |  |  | 316 | 12±3 | 0.12±0.05 | 5±1 | 0.12±0.05 | 4±1 | 0.13±0.07 |
|  |  |  | 1000 | 6.0±1.8 | 0.12±0.05 | 2.0±0.5 | 0.11±0.04 | 1.6±0.3 | 0.11±0.05 |
| 0.01 | 0.001 | 0.1 | 1 | 35±8 | 0.12±0.04 | 49±12 | 0.15±0.04 | 63±17 | 0.20±0.06 |
|  |  |  | 3 | 36±8 | 0.12±0.04 | 48±12 | 0.15±0.04 | 58±15 | 0.20±0.06 |
|  |  |  | 10 | 39±9 | 0.12±0.04 | 43±10 | 0.15±0.04 | 43±10 | 0.19±0.06 |
|  |  |  | 32 | 40±9 | 0.13±0.04 | 26±6 | 0.14±0.05 | 23±5 | 0.17±0.07 |
|  |  |  | 100 | 28±7 | 0.13±0.04 | 12±3 | 0.13±0.05 | 10±2 | 0.15±0.07 |
|  |  |  | 316 | 15±4 | 0.13±0.05 | 5±1 | 0.12±0.05 | 4±1 | 0.13±0.07 |
|  |  |  | 1000 | 7.1±2.0 | 0.12±0.05 | 2±1 | 0.11±0.05 | 1.5±0.3 | 0.11±0.05 |
| 0.1 | 0.001 | 0.01 | 1 | 27±7 | 0.13±0.04 | 24±7 | 0.14±0.05 | 19±6 | 0.16±0.07 |
|  |  |  | 3 | 27±7 | 0.13±0.04 | 23±7 | 0.14±0.05 | 18±6 | 0.16±0.07 |
|  |  |  | 10 | 27±7 | 0.13±0.04 | 21±6 | 0.13±0.05 | 16±5 | 0.16±0.07 |
|  |  |  | 32 | 25±7 | 0.13±0.04 | 16±4 | 0.13±0.05 | 11±3 | 0.15±0.07 |
|  |  |  | 100 | 19±6 | 0.13±0.05 | 9±2 | 0.13±0.05 | 6±2 | 0.14±0.07 |
|  |  |  | 316 | 12±3 | 0.12±0.05 | 4±1 | 0.12±0.05 | 3±1 | 0.12±0.07 |
|  |  |  | 1000 | 6.0±1.8 | 0.12±0.05 | 1.8±0.5 | 0.11±0.04 | 1.0±0.3 | 0.11±0.04 |
| 0.1 | 0.01 | 0.1 | 1 | 36±8 | 0.12±0.04 | 30±9 | 0.14±0.05 | 19±6 | 0.16±0.07 |
|  |  |  | 3 | 37±8 | 0.12±0.04 | 29±8 | 0.14±0.05 | 18±6 | 0.16±0.07 |
|  |  |  | 10 | 38±9 | 0.13±0.04 | 25±7 | 0.14±0.05 | 15±5 | 0.16±0.07 |
|  |  |  | 32 | 37±9 | 0.13±0.04 | 17±5 | 0.13±0.05 | 10±3 | 0.15±0.07 |
|  |  |  | 100 | 27±7 | 0.13±0.04 | 9±2 | 0.13±0.05 | 5±2 | 0.14±0.07 |
|  |  |  | 316 | 14±4 | 0.13±0.05 | 4±1 | 0.12±0.05 | 2±1 | 0.12±0.07 |
|  |  |  | 1000 | 6.9±2.0 | 0.12±0.05 | 2±1 | 0.11±0.04 | 1.0±0.3 | 0.11±0.04 |
| 1 | 0.01 | 0.01 | 1 | 21±6 | 0.13±0.05 | 8±3 | 0.12±0.05 | 4±1 | 0.12±0.05 |
|  |  |  | 3 | 21±6 | 0.13±0.05 | 8±3 | 0.12±0.05 | 3±1 | 0.12±0.05 |
|  |  |  | 10 | 20±6 | 0.13±0.05 | 7±3 | 0.12±0.05 | 3±1 | 0.12±0.05 |
|  |  |  | 32 | 19±6 | 0.13±0.05 | 6±2 | 0.12±0.05 | 3±1 | 0.12±0.05 |
|  |  |  | 100 | 16±5 | 0.13±0.05 | 5±1 | 0.12±0.05 | 2±1 | 0.11±0.05 |
|  |  |  | 316 | 10±3 | 0.12±0.05 | 3±1 | 0.11±0.05 | 1.1±0.4 | 0.11±0.05 |
|  |  |  | 1000 | 5.4±1.7 | 0.12±0.05 | 1.3±0.4 | 0.11±0.04 | 0.6±0.2 | 0.10±0.04 |
| 1 | 0.1 | 0.1 | 1 | 27±7 | 0.13±0.04 | 8±3 | 0.12±0.05 | 4±1 | 0.12±0.05 |
|  |  |  | 3 | 27±7 | 0.13±0.04 | 8±3 | 0.12±0.05 | 3±1 | 0.12±0.05 |
|  |  |  | 10 | 26±7 | 0.13±0.04 | 8±3 | 0.12±0.05 | 3±1 | 0.12±0.05 |
|  |  |  | 32 | 24±7 | 0.13±0.04 | 6±2 | 0.12±0.05 | 3±1 | 0.12±0.05 |
|  |  |  | 100 | 19±6 | 0.13±0.05 | 5±1 | 0.12±0.05 | 2±1 | 0.11±0.05 |
|  |  |  | 316 | 11±3 | 0.12±0.05 | 3±1 | 0.11±0.05 | 1.1±0.4 | 0.11±0.05 |
|  |  |  | 1000 | 5.8±1.8 | 0.12±0.05 | 1.3±0.4 | 0.11±0.04 | 0.6±0.2 | 0.10±0.04 |

**Table S6.** The average of kidneys and red marrow absorbed doses of ^177^Lu-labeled PSMA-specific ligands considering the different K_D_, k_off_, k_on_, λ_int_ and ligand amounts. ^a^K_D_=Dissociation constant (K_D_=k_off_/k_on_); ^b^k_off_ =Dissociation rate; ^c^k_on_=Association rate; ^d^λ_int_=Internalization rate.

| ***K_D_^a^***  **[nM]** | ***k_off_^b^***  **[1/min]** | ***k_on_^c^***  **[L/nmol/min]** | **Amount**  **[nmol]** | ***λ_int_^d^*=0.01 min^-1^** | | | ***λ_int_^d^*=0.001 min^-1^** | | | ***λ_int_^d^*=0.0001 min^-1^** | | |
| --- | --- | --- | --- | --- | --- | --- | --- | --- | --- | --- | --- | --- |
|  |  |  |  | **Ratio of absorbed dose**  **of…** | | | **Ratio of absorbed dose**  **of…** | | | **Ratio of absorbed dose**  **of…** | | |
|  |  |  |  | **Tumour 1 /Kidneys** | **Tumour 2 /Kidneys** | **Tumour REST /Kidneys** | **Tumour 1 /Kidneys** | **Tumour 2 /Kidneys** | **Tumour REST /Kidneys** | **Tumour 1 /Kidneys** | **Tumour 2 /Kidneys** | **Tumour REST /Kidneys** |
| 0.01 | 0.0001 | 0.01 | 1 | 1.4±1.0 | 1.7±1.3 | 1.1±0.9 | 1.5±1.0 | 1.7±1.4 | 1.2±0.9 | 1.5±1.0 | 1.7±1.3 | 1.2±1.0 |
|  |  |  | 3 | 1.4±1.0 | 1.7±1.4 | 1.1±0.9 | 1.5±1.0 | 1.8±1.4 | 1.2±1.0 | 1.5±1.1 | 1.7±1.3 | 1.3±1.0 |
|  |  |  | 10 | 1.5±1.0 | 1.7±1.4 | 1.2±0.9 | 1.6±1.1 | 1.9±1.5 | 1.3±1.1 | 1.7±1.2 | 1.9±1.5 | 1.5±1.1 |
|  |  |  | 32 | 1.6±1.1 | 1.9±1.5 | 1.3±1.0 | 2.1±1.5 | 2.4±1.9 | 1.8±1.4 | 2.4±1.7 | 2.5±1.9 | 2.2±1.6 |
|  |  |  | 100 | 2.1±1.5 | 2.3±1.9 | 1.7±1.4 | 3.0±2.2 | 3.2±2.5 | 3.1±2.2 | 3.0±2.1 | 3.2±2.3 | 3.7±2.6 |
|  |  |  | 316 | 2.7±1.9 | 3.0±2.3 | 2.7±2.0 | 3.3±2.3 | 3.6±2.6 | 4.2±2.8 | 3.1±2.1 | 3.3±2.3 | 4.2±2.6 |
|  |  |  | 1000 | 3.1±2.2 | 3.4±2.5 | 3.6±2.5 | 3.3±2.2 | 3.5±2.6 | 4.3±2.8 | 2.9±1.9 | 3.1±2.2 | 4.0±2.5 |
| 0.01 | 0.001 | 0.1 | 1 | 0.9±0.5 | 1.1±0.8 | 0.6±0.5 | 1.0±0.6 | 1.2±0.9 | 0.7±0.6 | 1.3±0.9 | 1.5±1.3 | 1.0±0.9 |
|  |  |  | 3 | 0.9±0.5 | 1.1±0.8 | 0.6±0.5 | 1.0±0.6 | 1.2±1.0 | 0.7±0.6 | 1.4±1.0 | 1.7±1.4 | 1.1±1.0 |
|  |  |  | 10 | 0.9±0.6 | 1.1±0.9 | 0.7±0.5 | 1.2±0.8 | 1.5±1.3 | 0.9±0.8 | 1.9±1.4 | 2.2±1.8 | 1.6±1.4 |
|  |  |  | 32 | 1.0±0.7 | 1.3±1.0 | 0.8±0.6 | 2.0±1.5 | 2.3±1.9 | 1.6±1.4 | 3.0±2.4 | 3.0±2.4 | 2.7±2.1 |
|  |  |  | 100 | 1.7±1.2 | 1.9±1.6 | 1.3±1.1 | 3.0±2.2 | 3.2±2.6 | 3.1±2.2 | 3.4±2.3 | 3.5±2.6 | 4.2±3.0 |
|  |  |  | 316 | 2.5±1.7 | 2.7±2.1 | 2.3±1.7 | 3.3±2.3 | 3.5±2.6 | 4.1±2.8 | 3.3±2.2 | 3.5±2.5 | 4.6±2.9 |
|  |  |  | 1000 | 2.9±2.0 | 3.2±2.4 | 3.3±2.3 | 3.2±2.2 | 3.5±2.5 | 4.3±2.7 | 3.1±2.1 | 3.2±2.3 | 4.3±2.7 |
| 0.1 | 0.001 | 0.01 | 1 | 1.5±1.0 | 1.7±1.4 | 1.2±0.9 | 1.8±1.3 | 2.1±1.6 | 1.6±1.2 | 2.4±1.7 | 2.6±1.9 | 2.4±1.7 |
|  |  |  | 3 | 1.5±1.0 | 1.7±1.4 | 1.2±0.9 | 1.9±1.3 | 2.1±1.7 | 1.6±1.2 | 2.5±1.7 | 2.7±1.9 | 2.5±1.8 |
|  |  |  | 10 | 1.5±1.0 | 1.8±1.4 | 1.2±1.0 | 2.0±1.4 | 2.3±1.8 | 1.7±1.3 | 2.7±1.9 | 2.8±2.1 | 2.8±1.9 |
|  |  |  | 32 | 1.7±1.1 | 1.9±1.6 | 1.4±1.1 | 2.5±1.8 | 2.7±2.1 | 2.3±1.7 | 3.2±2.2 | 3.3±2.4 | 3.6±2.4 |
|  |  |  | 100 | 2.1±1.5 | 2.3±1.9 | 1.8±1.4 | 3.3±2.3 | 3.5±2.7 | 3.5±2.5 | 3.5±2.4 | 3.6±2.6 | 4.7±3.1 |
|  |  |  | 316 | 2.8±1.9 | 3.0±2.3 | 2.7±2.0 | 3.5±2.4 | 3.8±2.8 | 4.5±3.0 | 3.3±2.2 | 3.5±2.5 | 4.8±3.0 |
|  |  |  | 1000 | 3.1±2.2 | 3.4±2.5 | 3.6±2.5 | 3.4±2.3 | 3.6±2.6 | 4.5±2.9 | 3.0±2.0 | 3.2±2.2 | 4.4±2.7 |
| 0.1 | 0.01 | 0.1 | 1 | 1.0±0.6 | 1.2±0.9 | 0.7±0.5 | 1.6±1.1 | 1.8±1.5 | 1.3±1.0 | 2.6±1.9 | 2.8±2.1 | 2.6±1.9 |
|  |  |  | 3 | 1.0±0.6 | 1.2±0.9 | 0.7±0.6 | 1.6±1.1 | 1.9±1.6 | 1.3±1.1 | 2.7±1.9 | 2.9±2.2 | 2.7±2.0 |
|  |  |  | 10 | 1.0±0.6 | 1.2±0.9 | 0.7±0.6 | 1.9±1.4 | 2.2±1.8 | 1.6±1.3 | 3.1±2.2 | 3.2±2.4 | 3.2±2.3 |
|  |  |  | 32 | 1.1±0.7 | 1.4±1.1 | 0.9±0.7 | 2.7±2.0 | 2.9±2.3 | 2.3±1.8 | 3.6±2.6 | 3.7±2.8 | 4.2±2.9 |
|  |  |  | 100 | 1.7±1.2 | 2.0±1.7 | 1.4±1.2 | 3.4±2.5 | 3.6±2.8 | 3.7±2.7 | 3.7±2.5 | 3.8±2.8 | 5.1±3.3 |
|  |  |  | 316 | 2.5±1.8 | 2.8±2.2 | 2.4±1.8 | 3.6±2.5 | 3.8±2.8 | 4.6±3.1 | 3.4±2.3 | 3.5±2.5 | 5.0±3.1 |
|  |  |  | 1000 | 3.0±2.0 | 3.2±2.4 | 3.4±2.3 | 3.4±2.3 | 3.7±2.7 | 4.6±3.0 | 3.1±2.0 | 3.2±2.2 | 4.5±2.7 |
| 1 | 0.01 | 0.01 | 1 | 1.8±1.3 | 2.1±1.7 | 1.5±1.2 | 3.0±2.1 | 3.2±2.4 | 3.1±2.2 | 3.1±2.1 | 3.3±2.3 | 3.9±2.5 |
|  |  |  | 3 | 1.8±1.3 | 2.1±1.7 | 1.5±1.2 | 3.0±2.1 | 3.3±2.4 | 3.2±2.2 | 3.1±2.1 | 3.3±2.3 | 4.0±2.5 |
|  |  |  | 10 | 1.9±1.3 | 2.1±1.7 | 1.6±1.2 | 3.1±2.1 | 3.3±2.5 | 3.3±2.3 | 3.2±2.1 | 3.3±2.3 | 4.1±2.5 |
|  |  |  | 32 | 2.0±1.4 | 2.3±1.8 | 1.7±1.3 | 3.3±2.3 | 3.5±2.6 | 3.7±2.5 | 3.2±2.2 | 3.4±2.4 | 4.3±2.7 |
|  |  |  | 100 | 2.4±1.7 | 2.6±2.1 | 2.1±1.6 | 3.6±2.5 | 3.8±2.8 | 4.4±3.0 | 3.2±2.2 | 3.4±2.4 | 4.6±2.9 |
|  |  |  | 316 | 2.9±2.0 | 3.2±2.4 | 3.0±2.1 | 3.6±2.5 | 3.9±2.8 | 4.9±3.2 | 3.0±2.0 | 3.2±2.2 | 4.4±2.7 |
|  |  |  | 1000 | 3.3±2.3 | 3.5±2.6 | 3.8±2.6 | 3.4±2.3 | 3.7±2.6 | 4.7±3.0 | 2.6±1.6 | 2.7±1.8 | 3.7±2.2 |
| 1 | 0.1 | 0.1 | 1 | 1.5±1.0 | 1.7±1.4 | 1.2±0.9 | 3.0±2.1 | 3.2±2.4 | 3.1±2.2 | 3.1±2.1 | 3.3±2.3 | 4.0±2.5 |
|  |  |  | 3 | 1.5±1.0 | 1.7±1.4 | 1.2±0.9 | 3.0±2.1 | 3.3±2.5 | 3.2±2.2 | 3.1±2.1 | 3.3±2.3 | 4.0±2.5 |
|  |  |  | 10 | 1.6±1.1 | 1.8±1.5 | 1.2±1.0 | 3.1±2.2 | 3.4±2.5 | 3.3±2.3 | 3.2±2.2 | 3.4±2.4 | 4.2±2.6 |
|  |  |  | 32 | 1.7±1.2 | 2.0±1.6 | 1.4±1.1 | 3.4±2.3 | 3.6±2.7 | 3.7±2.6 | 3.3±2.2 | 3.4±2.4 | 4.4±2.8 |
|  |  |  | 100 | 2.2±1.6 | 2.4±2.0 | 1.9±1.5 | 3.6±2.5 | 3.9±2.9 | 4.5±3.1 | 3.3±2.2 | 3.4±2.4 | 4.7±2.9 |
|  |  |  | 316 | 2.8±2.0 | 3.1±2.4 | 2.8±2.0 | 3.6±2.5 | 3.9±2.9 | 4.9±3.2 | 3.0±2.0 | 3.2±2.2 | 4.4±2.7 |
|  |  |  | 1000 | 3.2±2.2 | 3.5±2.6 | 3.7±2.5 | 3.4±2.3 | 3.7±2.7 | 4.7±3.0 | 2.6±1.6 | 2.7±1.8 | 3.7±2.2 |

**Table S7.** The average of tumour-to-kidneys absorbed doses of ^177^Lu-labeled PSMA-specific ligands considering the different K_D_, k_off_, k_on_, λ_int_ and ligand amounts. ^a^K_D_=Dissociation constant (K_D_=k_off_/k_on_); ^b^k_off_ =Dissociation rate; ^c^k_on_=Association rate; ^d^λ_int_=Internalization rate.

**Figure S2.** The absorbed dose for ^177^Lu 7.3 GBq in red marrow (no PSMA expression) of patient 5.
